# The effect of radiology services outsourcing on patients’ satisfaction in Tehran city hospitals

**Published:** 2015

**Authors:** H Mousavi, F Khodamoradi, CH Rostami Zarinabadi, H Mozafar Saadati, M Mohammadi, M Mahboubi, N Mousavi

**Affiliations:** * Kermanshah University of Medical Sciences, Kermanshah, Iran,; ** Tehran University of Medical Sciences, Tehran, Iran,; *** Ilam University of Medical Sciences, Ilam Iran,; **** Shahid Beheshti University of Medical Sciences, Tehran, Iran,; ***** Abadan School of Medical Sciences, Abadan, Iran,; ****** School of Engineering, Science and Research Branch, Islamic Azad University, Tehran,

**Keywords:** outsourcing, hospital, efficiency, effectiveness

## Abstract

**Background:** To have a developed society we should have healthy, active, and happy individuals and present that extended healthcare services perform an essential function in increasing the society’s health level. Health in a society includes the society’s, and people comfort with the condition and an assuring the situation that they can live healthily. Also, considering the self-governing plan of hospitals from 1995, the hospital authorities should choose a method of presenting services, and, the hospital is ready to present those activities during its own activities from an economic viewpoint. The current study was done while trying to discover the effect of the Outsourcing of the Radiology Unit on the patients’ satisfaction in hospitals of Tehran.

**Method:** The present research was done in a case-evidence and sectional study. Considering the weight of a month’s references to the Radiology Unit, which included around 1200 individuals, the volume of samples for measuring the frequency of the patients’ satisfaction with the means of Morgan table was similar to 291 individuals. To decrease the error percentage in each hospital we questioned 300 individuals. (n+10) were questioned and the gathered information were examined by what means SPSS application variant 21 and were then studied by climagraph – Smirnoph, Du Whitman – Vitney K tests.

**Findings:** the mean of the patients’ satisfaction of turned over and non-turned over Radiology Unit services were 41.46 and 45, respectively (from the maximum score of 60). A meaningful variation was seen among the patients'

Satisfaction in the two hospitals from the analytical viewpoint (p-value<0.001) and there was also a significant difference between the patients’ waiting time (p-value<0.001). The research’s findings showed that the outsourcing has a negative influence on the patients’ satisfaction and the duration of their waiting time.

**Conclusion:** Many times, managers do the outsourcing without considering individual and organizational dimensions and characteristics by just justifying it based on decreasing the expenses. Therefore, it is essential for authorities to consider not only the economic characters but also the individual and human aspects while setting the outsourcing contracts and arrangements.

## Introduction

Hospitals have a special importance as the greatest and very expensive operational unit of healthcare and treatment systems. Hospitals use 50-80 percent of all the expenses in the whole healthcare section and have a great contribution of educated and highest level of personnel [**1**]. 

The authority of government places a lot of pressure on the policymaking, execution, and observation of this section and leads the hospital system to turn over some of its administrative activities to the non-governmental section, in order to improve its efficiency [**2**]. Although, the procedure of variable the expenses and decreasing the resources is always increasing and the gap between the achievable and required resources is developing on a daily basis. What is more, is that the private hospitals, especially in expanding countries, which are directly governed by the state authorities, have weak performances, and efforts to improve their performance were not very efficient by applying internal management modifications [**3**]. During recent years, Iran has turned over a piece of the healthcare services to the private section aiming to better the modality of healthcare and treatment services, increasing the patients’ satisfaction and decreasing expenses [**4**]. Outsourcing includes the act of transferring some of the internal activities of an organization to its supplier outside of the organization and transferring the decision making right to the outside of the organization based on a contract. In fact, the outsourcing does not only imply activities but also manufacturing agents (human resources, equipments, facilities, technologies and other assets) and the authority of decision making in most cases is transferred [**5**]. 

Organizations try to turn over the internal affairs of the organization and make their body as small as possible due to various reasons [**6**]. Alvani believes that benefits resulted by outsourcing are factors such as decreasing expenses, organization’s concentration on its main activities, saving the moment for making the internal affairs of the structure, decreasing the risk by entering in partnership with another module in an unsafe business environment, improving consumer service, decreasing the company’s employees, creating the sense of competition in various sections of the organization [**7**]. Outsourcing was used to lead to an efficient management of the resources and increasing the modality and consent of various parties. Considering the existence of the facility of outsourcing in so many sections of the hospital, we could benefit from it in governing the hospital and we could evaluate the success rate by defining specific indexes [**8**]. The patients’ satisfaction is an important scale to evaluate services or received product because satisfied patients are more eager to continue using medical and healthcare services, keeping their contact with the service supplier and following the medical and control regimes [**9**]. Con Vikticle et al. defined the care quality as the satisfying of physical needs with providing professional care, social-mental support, satisfaction with care [**10**] and ensuring the presence of general and multi-dimensional cares to the patient [**10**,**11**]. Many expertises consider the patients’ rate of consent from the hospital aims as important indexes of efficiency and aim modality in various sections [**12**]. It shows that studying the patients’ consent is important outputs of the healthcare systems [**13**,**14**] and evaluating the care by the patient is also one of the major methods to measure and scale the quality of medical and healthcare services [**15**]. Moreover, Peiravi also mentioned in his research that in Iran, the Ministry of Health has obligated all hospitals to do periodic evaluations regarding the patients’ satisfaction and also required interventions to increase the patients’ satisfaction, since 2011, so that it can comply with its main mission [**16**]. Considering the mentioned issues about the patients’ satisfaction, we should also point out the important issue that although nowadays companies move toward outsourcing, it represents a part of their liability in the world, so that they can achieve benefits such as decreasing expenses to get hold of advanced technology. Nevertheless, the difference in organizational cultures and complexity of managing created relations means that outsourcing could guide to failure or lack of satisfaction [**17**]. Therefore, all the above-mentioned issues confirmed that we should select a method that the present services to the patients should work with, having the lowest expense and highest quality, so that the hospital’s expenses could be minimized on one hand and the hospital should be enabled to continue working and present the highest quality services to the patients on the other hand. This way, not only their expenses decrease but they also achieve their satisfaction. Also, this study was executed while aiming to research the influence of outsourcing on the efficiency and affectivity of Radiology Unit services in Hospitals of Tehran City. 

## Method 

This study was an analytical and witness based study, its results being applicative, and its time duration being periodic, during the time period of June until January 2013. The current study was done in the Educational-Medical Hospitals of Tehran. The statistical society included all the patients during a month in the Radiology Unit in both hospitals (N=1200). Considering the fact that a load of people coming to the Radiology Unit was around 1200 individuals in any hospital, the case bulk to measure the patients’ consent rate was calculated to be equal to 291 individuals by means of Morgan table. 300 individuals (n+10) were questioned in each hospital to lower the error percentage and the simple accidental sampling method was used for sampling. The method to gather data for the study of the radiology patients’ consent was the field method and its tool was a questionnaire. This questionnaire consisted twelve asks concerning the condition of the unit, 2 related to the waiting duration. The mentioned questionnaire was scaled via Likert scale from 1 to 5 in such a manner that number 1 was relevant to the lowest level of satisfaction and number 5 was related to the great amount of satisfaction, the maximum point being 60 points. Questions relevant to the waiting time duration were separated to 2 sections. The first question was relevant to the time between the entrance and admission of the sick mans and another question was related to the time between the admission and receiving radiology services. The questionnaire was designed by Medical University lecturers and its admissibility–stability was also confirmed by lecturers and also hospitals clinical governance committee. To confirm the credibility and admissibility, the questionnaire was analyzed and confirmed by 10 experts. What is more, is the reality that to specify the consistency of the questionnaire, Cronbach alpha was applied. After gathering the research data, finally, the gathered data were excavated by SPSS copy 21 and, to excavate data, descriptive statistics was used in redundancy, median, standard deviation tables, and deductive statistics including Kelmogroph–Smirnoph test, Uman–Whitney test and K2. 

## Findings 

Findings of the present research indicated that the median of the patients’ consent in the turned over module was equivalent to 41/ 46 (7/ 3±) from the maximum 60 points, equal to (69%) and the median of the patients’ satisfaction in the non-turned over unit was equal to 45 (6/ 94±) from the highest mark of 60 points (75%). This fact indicated a lower satisfaction of pointers in the turned over section in comparison with the general part, and, the minimum medians among all aspects, was relevant to the patients’ consent in turned over Radiology Units, also being related to giving turns to the system with the lowest median of 3.11, satisfaction of the existing comforting equipments in the unit with a median of 3.36, and personnel behavior while getting accepted, 3.41. 

**Fig. 1:**
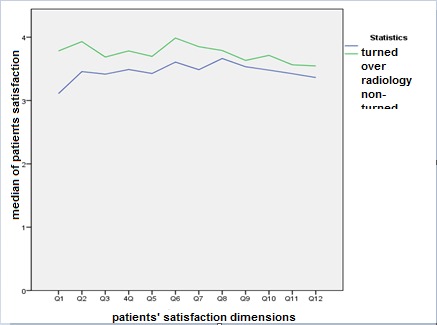
Median comparison of any dimension of the patients’ consent in both hospitals

In order to determine the norm of distributing information relevant to the patients’ consent the climagraph-Smirnoph exam was applied. By means of this test, it was determined that the generalization mark of the patients’ consent was not normal (p-value=0.000). Also, to compare the median scores in the two units, nonparametric Uman-Vitney test for the patients’ consent score was used. A significant difference was observed between the patients in these two hospitals (p-value=0.000) and since the p-value was so small, it clearly showed the severity of differences among medians. Therefore, it was obvious that the outsourcing had a great impact on the patients’ consent. 

Also, the results indicated that most patients were accepted in a time duration of 16 to 20 minutes after entering the unit in turned over units, while in non-turned over units, most of the patients (42%) were accepted in 5 to 10 minutes. Findings also showed that most patients (42%) of turned over units, were X-rayed in a 16-20 minutes time period, after they were settled, while in non-turned over units, most patients (33.3%) were X-rayed 5-10 minutes after they were received. Time duration patients waited for to be received in two units, showed a significant difference based on K2-test (p-value-0.000) and the waiting time in-between, and the X-ray in both units was less significant based on K2-test (p-value -0.000). Since the p-value was so small in both cases, it indicated that the outsourcing had a great influence on the patients’ waiting time duration since they entered, were received, and were X-rayed. Patients’ waiting time duration is presented in **Table 1**. 

Results also indicated that 225 individuals among patients of turned over unit (75%) and 277 individuals between the sick mans of the non-turned over unit (92.3%) category suggested these modules to anthers and this disagreement was significant based on the J2-test (p-value-0.000). Therefore, the outsourcing had a great impact on suggesting the Radiology Unit to other patients. 

**Table 1:** Redundancy percentage of expecting moment duration between the patients’ entrance and being received

			Time duration between entrance and being received						
			10- 5	15 - 11	20 - 16	30 - 21	45 - 31	More than 45	Total
Hospital	Firuzgar	number	25	73	129	60	12	1	300
		percentage	8.3%	24.3%	43.0%	20.0%	0.4%	0.3%	100.0%
	Valiasr	number	126	104	54	11	2	3	300
		percentage	42.0%	34.7%	18.0%	3.7%	0.7%	1.0%	100.0%
Total		number	151	177	183	71	14	4	600
		percentage	25.2%	29.5%	30.5%	11.8%	2.3%	0.7%	100.0%

**Table 2:** Redundancy percentage of time duration between being received and radiography

			Time duration between being received and radiography						
			10- 5	15 - 11	20 - 16	30 - 21	45 - 31	More than 45	Total
Hospital	Firuzgar	number	19	58	121	66	23	13	300
		percentage	6.3%	19.3%	40.3%	22.0%	7.7%	4.3%	100.0%
	Valiasr	number	100	91	63	28	9	9	300
		percentage	33.3%	30.3%	21.0%	9.3%	3.0%	3.0%	100.0%
Total		number	119	149	184	94	32	22	600
		percentage	19.8%	24.8%	30.7%	15.7%	5.3%	3.7%	100.0%

Findings resulted in this current research, displayed that the median of other sectors authorities’ satisfaction with the turned over unit of 29.26 (5.65±) had the maximum score of 70 points and the median of other sectors authorities’ satisfaction of 28.97 (5.1±) had a maximum score of 70 points. The authorities’ satisfaction with the turned over section and non-turned over section was almost equal. The lowest median observed among items related to the authorities’ satisfaction with a turn over radiology unit was related to question number 3 in relation with on time access to portable radiology devices with the minimum median of (2.72) and accepting suggestions and also applying them by the median of (3.05). 

## Discussion 

The present research was done aiming to determine the influence source of out on the output and affective of hospitals Radiology Unit. Finding obtained to that the weight media of patients’ satisfaction score in turn over radiology units was (41.46) and in non-turned over was (45), being significant from the difference statistical point of view. Results indicated that the patients’ consent was higher with non-turned over radiology units and therefore the outsourcing had no positive affect on the patients’ satisfaction which was similar to studies of Baderaldin, Amerion and Mohaghegh and in contrast with primarily expectancy of executing outsourcing procedure. The most extended rate of discontent between patients in analyzing the major factors of discontent was the long time duration of waiting [**18**]. Amerion considered the lack of sufficient explanation in order to get ready for radiology the reason for the minimum rate of satisfaction (7.8%) [**14**], while Mohaghegh et al. presented the insufficient time investment of pharmacy employees for consulting [**4**] as being a reason. Meanwhile, the present research daresay that loss consent in turned over unit was the conclusion of giving the turn system the minimum median of (3.11) and, to confirm this, we could mention the lack of devoting enough work force for recipient part in turned over unit, existence of 5 secretaries in 3 working shifts for the whole unit, which included radiology, MRI, bone accumulation and another aims, led to the increment in work load, and, as a finding, caused problems in the system of giving turns. 

The second factor, which had the lowest rate of satisfaction, was relevant to the welfare equipments, which was observed in both turned over and non-turned over units. The mentioned median was equivalent to (3.36) in non-turned over unit and this was more because of loss work force to move the wheelchair, while this median was equivalent to (3.54) in the turned over unit due to the lack of equipments inside the unit. 

The third factor that showed the lowest median in analogy with the other factors in turned over units was the individual’s behavior while receiving the patient, with a median equal to (3.41) and such a claim could be justified by the reception of the personnel’s high work load and their exhaustion. Therefore, to fix this problem, not only should we decrease workload but it is also necessary to educate the critical communication skills to turn over units’ personnel. 

The results of the present research also indicated that most of the patients (43%) were received in a time duration of 16-20 minutes while the receiving time duration in non-turned over for most of the patients (42%) was equal to 5-10 minutes after entering the unit, which showed a significant statistical difference. Badroldin concluded in a research entitled “Patients’ satisfaction with medical services in educational Saudi Arabia hospital” that the most important factor expressed by patients about their dissatisfaction with medical services was the long time duration of waiting to receive their medications [**18**]. Moreover, Amerion found out a study entitled “Outpatients and inpatients’ satisfaction of army hospitals” that the largest dissatisfaction (19.2%) in the pharmaceutical sector, was the long waiting time to receive medication, and the minimum rate of dissatisfaction (19.2) in clinic, was relevant to the reality that doctors made patients wait and generally, most of significant reason of dissatisfaction in current study, was related to the waiting time to receive medication (19.2%) [**14**]. 

By the present research, we could consider that increasing the time duration to receive a patient is because of a loss enough workforce in the reception and, increasing the waiting time to be scanned is because of loss of enough workforce in the scanning section. Therefore, obtained results indicated a negative influence of outsourcing on the patients’ waiting time duration. 

What is more important is the fact that based on obtained results, 225 individuals among the patients who went to turned over radiology (75%) and 277 individuals among the patients who went to non-turned over radiology (92.3%) recommended these hospitals to others and the upper percent of non-turned over radiology indicated the higher rate of patients’ satisfaction with these types of units. 

## Conclusion 

The present research explained that the rate of patient’s satisfaction with radiology services was decreased and this showed that managers usually turn over services without any consideration related to human and organizational characteristics and dimensions, justifying it by decreasing costs. So, it is necessary and critical for authorities to consider not only the financial aspects but also the individual and human aspects of the procedure while settling an outsourcing contract.

**Acknowledgements**

This research is the conclusion of project as **“The effect of radiology services outsourcing on patients’ satisfaction in Tehran city hospitals”** approved by the Student Research council, Medical Science Kermanshah University in 1394 with the code 94027.
